# The radiosensitizing effect of doranidazole on human colorectal cancer cells exposed to high doses of irradiation

**DOI:** 10.1186/1471-2407-7-188

**Published:** 2007-10-06

**Authors:** Li Zhang, Aimin Gong, Jun Ji, Yuanyuan Wu, Xiaoyu Zhu, Suqing Lv, Hongzhu Lv, Xizhuo Sun

**Affiliations:** 1 Department of Central Laboratory, Dalian Municipal Central Hospital, Xinan-Road 826, Dalian, 116033, China; 2 Xinhua Hospital, Dalian University, Xinhua street 156, Dalian, 116021, China

## Abstract

**Background:**

This paper investigates the effects of a new radiosensitizer, doranidazole, and enhancing irradiation on colorectal cancer cells.

**Methods:**

The radiosensitizing effect of doranidazole was determined using colony formation and propidium iodide (PI) assays to measure cell growth inhibition and the cell killing effect of human colorectal cancer cell lines exposed to high doses of γ-ray irradiation under hypoxic conditions *in vitro*. Fluorescence staining and cell migration assays were also used to assess the radiosensitizing effect.

**Results:**

Cell proliferation evaluated by clonogenic survival curves was significantly inhibited by 5 mmol/L doranidazole, particularly at doses ranging from 10 to 30 Gy of irradiation. The radiosensitizing effect of doranidazole on colorectal cancer cells occurs in a time- and dose-dependent manner. Doranidazole also inhibited the mobility of cell invasion and migration.

**Conclusion:**

Doranidazole can enhance the killing effect and the cell growth inhibition of colorectal cancer after high-dose irradiation in a time and dose-dependent manner.

## Background

Colorectal cancer is the fourth most common malignant tumor worldwide [1]. Local recurrence is a challenge in the treatment of colorectal cancer, since it disables the transference and it is difficult to treat [2]. The incidence of local recurrence ranges from 15 to 45 percent after conventional surgery, in which blunt dissection of the colorectal fascia often fails to remove all of the tissue that may bear a tumor [3]. In an attempt to improve local control and survival after conventional surgery, radiotherapy is one of the major adjuvant therapies for colorectal cancer. Preoperative radiotherapy and postoperative radiotherapy might reduce the risk of local recurrence and improve survival rates [4]. A recent meta-analysis concluded that the combination of preoperative radiotherapy and surgery, as compared with surgery alone, significantly improved overall survival and cancer-specific survival [5,6]. However, the radiation response is limited because of the presence of radio-resistant hypoxic cells within solid tumors [7].

Several members of the family of hypoxic radiosensitizers have been extensively explored over the past 30 years [8], and nitroimidazole derivatives are major compounds examined in clinical studies [9]. However, most clinical trials have failed to document a significant sensitizing effect [10,11]. In these clinical studies, low doses of drugs were used repeatedly with fractionated irradiation because of their side effects, such as neurotoxicity.

Doranidazole, a 2-nitroimidazole-nucleoside derivative, was synthesized to reduce the lipophilicity of nitroimidazole derivatives and to prevent the accumulation of the drug in the central and peripheral nervous systems [12]. Doranidazole can be given at high doses during intraoperative high-dose irradiation. It has been shown that doranidazole suppresses the re-growth of a subcutaneously implanted mouse tumor after 20 Gy irradiation [13].

In our previous study, we reported the radiosensitizing effect of doranidazole on human pancreatic cancer cells after high-dose irradiation under hypoxic conditions [14]. In the present study, we examined the radiosensitizing effect of doranidazole on human colorectal cancer cells by high-dose irradiation in vitro.

## Methods

### Cells and cell culturing conditions

The five human colorectal cell lines used in this study: HT-29, DLD-1, Colo 201, LoVo, and SW620, were obtained from the Japanese Cancer Research Resources Bank (Tokyo, Japan). Cells were maintained in Dulbecco's Modified Eagle's Medium (DMEM, Sigma Chemical Co., St. Louis, MO) supplemented with 10% fetal bovine serum (FBS), streptomycin (100 mg/mL), and penicillin (100 u/mL) at 37°C in a humidified atmosphere containing 95% air and 5% CO_2_. The experiments were performed 24 h after plating cells in Falcon flat-bottom 24-well plates (Becton-Dickinson, Lincoln Park, NJ) during logarithmic growth. All of the procedures were performed in triplicate, and each was repeated at least three times.

### Chemicals

Doranidazole, provided by POLA Chemical Industry Inc. (Yokohama, Japan), was dissolved in phosphate-buffered saline (PBS). Cells were treated with doranidazole for 1 h during irradiation at various concentrations (indicated below). After irradiation, the cells were washed with PBS and fresh medium was added. Anticancer agents, VP-16, 5-FU, SN-38, and cisplatin were provided by Sigma Chemical Co.. The anticancer agents were applied to cells continuously during the entire experimental period.

### Irradiation

Cells in 24-well plates were irradiated with three different doses (10, 20 and 30 Gy) at room temperature with a ^137^Cs source (Gamma Cell 40, Atomic Energy of Canada Ltd., Mississauga, Canada) delivering 1.0 Gy/min. During irradiation, hypoxia was maintained in cells by placing plates in a closed chamber and flushing with 100% N_2 _30 min prior to the treatment, as described previously [14].

### Cell death detection

We assessed the radiosensitizing effect of doranidazole on the colorectal cancer cells by determining the extent of cell death by the fluorescence intensity of PI stained cells, as described previously [5]. Briefly, PI was added to each well to a final concentration of 30 mmol/L, and the initial fluorescence intensity from the dead cells was measured in a multi-well plate reader, VICTOR 3 1420 Multi-label Counter (Perkin Elmer, Wallac Oy Inc., Turku, Finland) with 530-nm excitation and 645-nm emission filters. Digitonin (600 nM) was added to each well to permeabilize all cells and label all nuclei with PI following the initial assessment of intensity. After a 30 min incubation, the fluorescence intensity was measured again to obtain a value corresponding to the total number of cells. The percentage of dead cells was calculated as the proportion of the initial fluorescence intensity over that corresponding to the total number of cells.

### Colony forming assay and cell survival curve

The number of colonies was determined as described previously [16]. Briefly, after treatment with irradiation and doranidazole, cells were trypsinized, counted, and seeded for the colony forming assay in 60 mm dishes at 300 cells per dish. After incubation for 14 days, colonies were stained with crystal violet and the number of positive cells was counted. Colonies containing more than 50 cells were scored, and triplicates containing 10–150 colonies/dish were counted in each treatment.

In both of the experiments performed under hypoxic condition, the cell surviving fraction was determined by a standard in vitro colony formation assay and cell survival curve. The sensitizer enhancement ratio (SER) was calculated from two radiation doses with or without doranidazole to reduce cell survival to 1%.

SER = irradiation D0/(irradiation + doranidazole) D0

### Morphological observation with fluorescence microscopy

The extent of cell killing via apoptosis was assessed by fluorescence microscopy, using the nuclear fluorophore Hoechst 33258 (Ho258) and a Leica inverted microscope (DM IRB, Leica, Germany). The cells were cultured in 24-well plates and observed 4 days after treatment with irradiation and/or doranidazole. Thirty minutes before viewing, the cells were treated with 5 mmol/L Ho258. Images were acquired using a Spot-2 cooled CCD digital camera (DC300F, Leica) and 365–395 nm excitation, with an emission range of 435–485 nm.

### Cell migration and invasion assays

Cell matrigel invasion and migration assays were performed as previously described [17]. A total of 5 × 10^4 ^cells in 500 μL of serum-free DMEM were added to the upper chamber of Boyden chambers (Transwell, 6.5 mm diameter, 8 μm pore filter; Costar, Cambridge, MA) and allowed to migrate for 24 h at 37°C under cell culture conditions. DMEM (500 mL) containing 10% FBS was added to the bottom chamber and used as a chemo attractant. Cells were removed from the upper chamber with a cotton swab and the cells that migrated to the lower surface of the membrane were fixed, stained with hematoxylin and eosin (H&E) and counted.

### Statistical analysis

For the cell survival assay, experimental procedures were performed in triplicate, and the results were expressed as the mean ± SD from three or four independent experiments. Statistical significance was analyzed with the Mann-Whitney t-test using Statview software (JMP, Cary, NC, USA) *P *< 0.05 was considered to be statistically significant.

## Results

### The effect of doranidazole on cell viability and killing in a human colorectal cancer cell line, Colo 201

**Figure 1:**
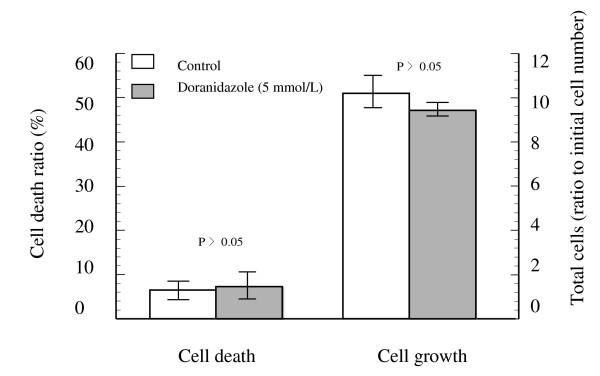
Cell growth and cell death of Colo 201 exposed to Doranidazole for 4 days.

To determine the cytotoxicity of doranidazole on human colorectal cancer cells, we examined Colo 201 cells by PI assay (as described in the *Materials and Methods*). As shown in Figure 1, doranidazole alone did not cause any cell growth or a cell killing enhancement effect at 5 mmol/L under either aerobic or hypoxic conditions (data not show).

### Doranidazole radiosensitizes the cell growth viability of Colo 201 cells under hypoxic conditions

**Figure 2:**
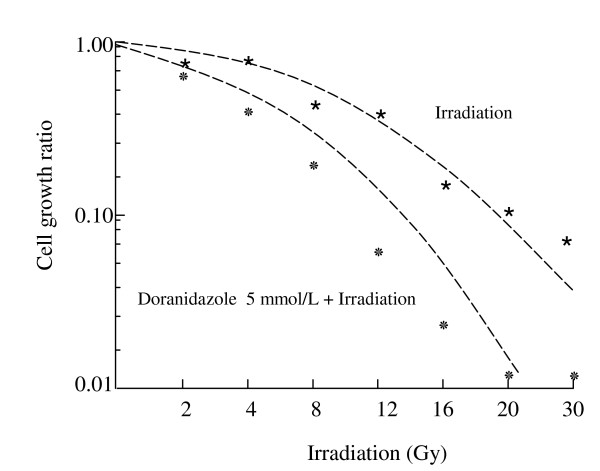
Cell survival curve of Colo 201 after different doses of irradiation under hypoxia in the presence or absence of 5 mmol/L Doranidazole.

Treated with and without doranidazole during irradiation, Colo 201 cells were irradiated under hypoxic conditions for 1 h at doses of 2, 4, 8, 12, 16, 20 and 30 Gy. The cell growth ratio was determined by colony assay 2 weeks after treatment. As shown in Figure 2, cell proliferation was affected by irradiation alone in a dose-dependent manner; in contrast, the addition of 5 mmol/L doranidazole significantly reduced the cell survival rate under hypoxic conditions. The SER of doranidazole was determined for Colo 201 cells after irradiation under hypoxic conditions. The SER of doranidazole for low dose irradiation (2 – 10 Gy) was 1.26, and that for high dose irradiation (10–30 Gy) was 1.79.

### The enhancement effect of doranidazole on cell killing under hypoxic conditions after high-dose irradiation in colorectal cancer cell lines

**Figure 3:**
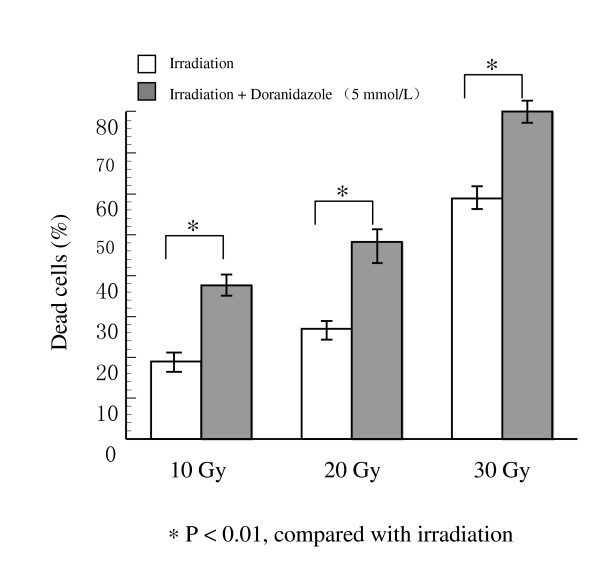
Killing of a human colorectal cancer cell line, Colo 201, exposed to different doses of irradiation under hypoxia.

The effect of doranidazole was examined in different cell samples following treatment with different doses of irradiation. As shown in Figure 3, 4 days after the irradiation, the percentage of dead cells was augmented and correlated with increasing doses of irradiation: 18.5% with 10 Gy, 27.8% with 20 Gy, and 57.9% with 30 Gy. The addition of 5 mmol/L doranidazole significantly increased the cell death by approximately 85%, 70%, and 30%, respectively. Similarly, with the addition of 5 mmol/L doranidazole, the cell killing by 30 Gy irradiation under hypoxic conditions was significantly increased from 22.2% to 36.4% in five colorectal cancer cell lines (Table 1).

**Table 1:** The effect of doranidazole on human colorectal cell lines 3 days after irradiation under hypoxia

Irradiation	Dead Cells (%)				
	
	VoLo	HT-29	DLD-1	Colo 201	SW 620
30 Gy irradiation	15.7 ± 1.6	21.9 ± 3.7	16.7 ± 1.9	12.7 ± 2.4	19.5 ± 0.9
30 Gy irradiation + Doranidazole (5 mM)	22.2 ± 2.9 *	33.8 ± 4.0 *	28.9 ± 3.5 *	31.4 ± 1.9 *	27.0 ± 1.9 *

* *P *< 0.01, compared with 30 Gy irradiation

### Time-course experiments confirmed the enhancing effect of doranidazole in Colo 201 cells

**Figure 4:**
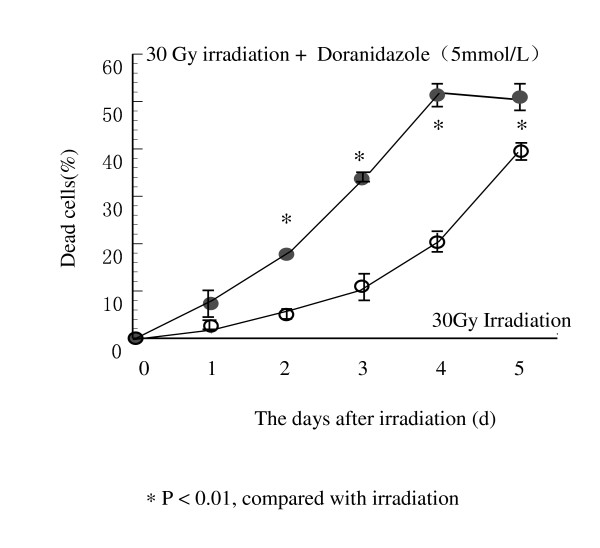
Serial measurement of cell death of human colorectal cancer cell line, Colo 201, after 30 Gy irradiation under hypoxia in the presence or absence of 5 mmol/L doranidazol.

As shown in Figure 4, significant cell killing was found 3 days after 30 Gy irradiation, and the percentage of cell killing was significantly increased in the presence of 5 mmol/L doranidazole 2 days following the irradiation.

A dose-dependent analysis of doranidazole revealed that cell killing with 30 Gy irradiation increased with increasing doses of doranidazole, and a statistically significant increase in cell death was observed at doses greater than 5 mM (data not shown), as published previously for other tumor cells (14).

### Morphological changes of Colo 201 cells treated with doranidazole after 30 Gy irradiation under hypoxia

**Figure 5:**
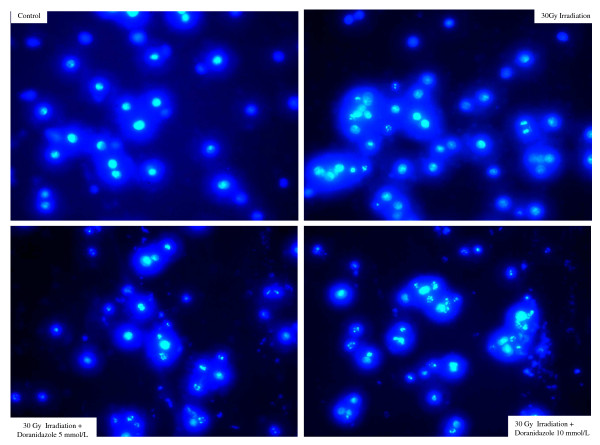
The nuclear morphologic changes of Colo 201 cells after irradiation under hypoxia condition in the presence or absence of Doranidazole.

Four days after irradiation, the incubated Colo 201 cells were stained with Ho258 and observed using a microscope. As shown in Figure 5, the cells irradiated with 30 Gy irradiation showed damage including swelling, membrane dissolution and formation of debris. Some cells also showed nuclear fragmentation, which is a manifestation of apoptosis. These damage effects were aggravated as the concentration of doranidazole increased from 5 to 10 mmol/L.

### Analysis of the cell migration assay of Colo 201 cells after irradiation

**Figure 6:**
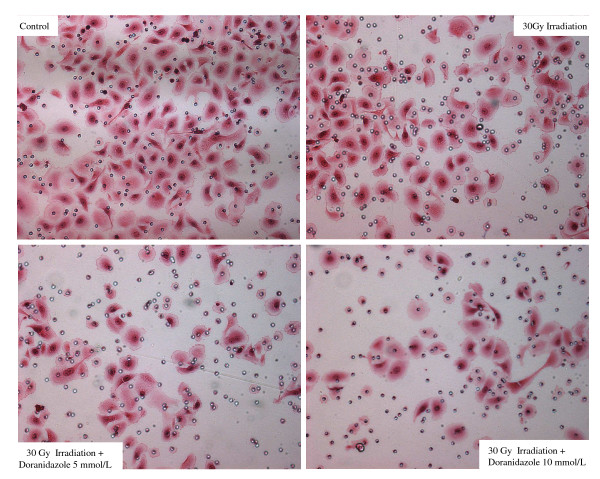
The cell migration assay of human colorectal cancer cell line, Colo 201 after irradiation under hypoxia condition in the presence or absence of doranidazole.

The number of cells that migrated to the lower surface of the membrane 24 h after 30 Gy irradiation was significantly lower than that of the control cells. Furthermore, the number of cells that had migrated was significantly reduced by doranidazole in a dose-dependent manner (Figure 6).

### The radiosensitizing effects of four anticancer agents on Colo 201 cells compared to those of doranidazole

Using 30 Gy irradiation with VP-16, cisplatin, SN-38, 5-FU or doranidazole, we increased the rate of cell killing significantly compared to that with irradiation alone. Treatment with the anticancer agents alone at the radiosensitizing doses significantly killed Colo 201 cells, which contrasted with the noncytotoxic effects of doranidazole (Table 2).

**Table 2:** Effect of doranidazole and anticancer agents on the cell killing of Colo 201 cell exposed to 30 Gy irradiation under hypoxia

Drugs	Dead cells (%)	
	
	No-irradiation	30 Gy irradiation
Control	0.2 ± 0.3	18.4 ± 3.4
Doranidazole (5 μM)	0.8 ± 6.3	35.8 ± 4.4 *
VP-16 (5 μM)	24.4 ± 1.7	36.9 ± 1.2 *
Cisplatin (10 μM)	27.1 ± 0.3	38.1 ± 3.5 *
SN-38 (10 μM)	21.2 ± 6.7	37.4 ± 9.1 *
5-FU (50 μM)	31.5 ± 1.8	47.6 ± 2.7 *

* *P *< 0.01, compared with 30 Gy group

## Discussion

The results of the present study clearly demonstrate that doranidazole enhances the growth inhibition and killing of human colorectal cancer cells exposed to high-dose irradiation under hypoxic conditions. The results were confirmed by cell growth and cell killing assays, apoptotic analysis, morphologic observations, and matrigel invasion and migration assays. The cell killing doses of irradiation adopted in the present study are comparable to those used in clinical practice during intraoperative irradiation for the treatment of advanced colorectal cancer. The doses of doranidazole used in the present experiment are achievable in patients when administered intravenously, and have been demonstrated previously in pancreatic cancer cells [14]. Taken together, doranidazole may function as a potential sensitizer of intraoperative radiotherapy for advanced colorectal cancer.

As a radiosensitizer, the clinical benefit of doranidazole is different from that of anticancer agents. As shown in the present study, anticancer agents not only enhance the cell killing resulting from high dose irradiation, but they also kill cells without irradiation. At radiosensitizing doses anticancer agents have adverse effects on non-irradiated organs, including the bone marrow and intestines. In contrast, treatment of cells with doranidazole alone did not cause any cytotoxic effects to either normal fibroblasts or malignant colorectal cancer cells. Meanwhile, systemically administered doranidazole will cause the enhancing effects only on the irradiated tumor and will not harm the surrounding non-irradiated normal tissues.

The radiosensitizing effects of doranidazole on cell killing and cell growth inhibition may facilitate the success of pre-operative or post-operative irradiation in cases of advanced colorectal cancer, particularly by enhancing the inhibition of cancer cell metastasis of colorectal cancer after irradiation. Therefore, doranidazole and intraoperative irradiation will reduce the incidence of local recurrence after the conventional surgery for colorectal cancer.

In a previous study, we reported the radiosensitizing effect of doranidazole on human pancreatic cancer cells [14]. In the present study, we confirm the radiosensitizing effect of doranidazole on human colorectal cancer cells. The results clearly indicate that doranidazole may reduce the risk of local recurrence and improve survival rates after the combination of pre-operative or intraoperative radiotherapy and surgery. Taken together, doranidazole will work as a potential sensitizer of radiotherapy for human colorectal cancer.

## Competing interests

The author(s) declare that they have no competing interests.

## Authors' contributions

J Ji carried out the majority of studies, and participated in cell culture and cell analysis. YY Wu and XY Zhu participated in the design of the study and performed the statistical analysis. SQ Lv and HZ Lv carried out the irradiation. L Zhang and AM Gong conceived of the study, drafted the manuscript and participated in its design. XZ Sun conceived of the study and participated in its coordination. All authors read and approved the final manuscript.

## Pre-publication history

The pre-publication history for this paper can be accessed here:


